# Targeting endoplasmic reticulum stress and protein misfolding in schizophrenia: the emerging promise of sigma-1 receptor agonists

**DOI:** 10.1007/s00213-025-06940-6

**Published:** 2025-11-11

**Authors:** Mariam K. Ahmed, Kareem Abdou, Weam W. Ibrahim, Ahmed F. Mohamed, Noha A. El-Boghdady

**Affiliations:** 1 Department of Biochemistry, Faculty of Pharmacy, Cairo University, Kasr El Aini St., Cairo, 11562 Egypt; 2 Department of Pharmacology and Toxicology, Faculty of Pharmacy, Cairo University, Cairo, Egypt; 3 Faculty of Pharmacy, King Salman International University (KSIU), South Sinai, 46612 Egypt

**Keywords:** Schizophrenia, Endoplasmic reticulum stress, Sigma-1 receptor, Protein misfolding, Unfolded protein response

## Abstract

Schizophrenia is a severe psychiatric disorder marked by significant cognitive, perceptual, and social deficits, the neurobiological basis of which remains incompletely elucidated. Increasing evidence implicates disruptions in protein homeostasis, including misfolding and aggregation of key neuronal proteins, as contributing factors to its pathogenesis. While proteinopathies have been extensively studied in neurodegenerative diseases, their role in schizophrenia has only recently gained attention. Central to these processes is endoplasmic reticulum (ER) stress and the activation of the unfolded protein response, which regulate protein folding and cellular quality control. Dysregulation of ER stress pathways, alongside impaired chaperone protein function and mitochondrial dysfunction, can lead to accumulation of misfolded proteins and neuronal dysfunction. Proteins such as DISC1, CRMP1, NOS1AP, and others have been identified with altered expression and aggregation patterns in schizophrenia, linking protein abnormalities to disease pathology. Additionally, mounting evidence suggests that chronic ER stress can activate microglia, the brain’s immune cells, triggering the release of proinflammatory cytokines and promoting neuroinflammation. Sigma-1 receptor, a unique ER chaperone protein involved in modulating ER stress and calcium signaling, has emerged as a critical regulator of neuronal proteostasis and survival. Agonists of the sigma-1 receptor show promising therapeutic potential by alleviating ER stress, enhancing neuroprotection, halting inflammation, and restoring cellular homeostasis in preclinical models of schizophrenia and other brain disorders. In this review, we will discuss these interconnected molecular mechanisms, highlighting novel therapeutic pathways focused on proteostasis restoration and sigma-1 receptor modulation, which offer a promising avenue for future interventions in schizophrenia.

## Introduction

Schizophrenia is a chronic and severely debilitating psychiatric condition marked by a range of symptoms that substantially affect an individual’s cognition, emotions, and conduct **(**Singh and Chaudhuri 2014**)**. It is characterized by a range of symptoms including positive psychotic symptoms (hallucinations, delusions, disorganized or catatonic speech and behavior); negative symptoms (diminished motivation and expressiveness, asociality, anhedonia, and avolition); and cognitive symptoms (deficient executive function, impaired short-term memory, and decreased mental processing speed) **(**Jauhar et al. 2022). A significant percentage of people with schizophrenia display pronounced negative symptoms (60%) and cognitive symptoms (80%). Cognitive impairment associated with schizophrenia (CIAS) significantly leads to long-term chronic disability and correlates with more severe functional outcomes **(**Terry-Lorenzo et al. 2025). First-generation antipsychotics, such as haloperidol and chlorpromazine, exert their therapeutic effects by blocking dopamine D₂ receptors and remain effective in treating positive symptoms of schizophrenia. However, their therapeutic application is limited due to a high rate of extrapyramidal side effects **(**Abou-Setta et al. 2012; Nasim et al. 2025). Second-generation antipsychotics, such as risperidone, olanzapine, quetiapine, and clozapine, target both dopamine D₂ and serotonin 5-HT₂A receptors, providing efficacy against both positive and, to some extent, negative symptoms, with a comparatively lower risk of motor side effects but a higher metabolic burden **(**Kanani and Pillai 2020; Weston-Green 2022**)**. Despite these advances, currently approved pharmaceutical agents primarily target dopamine and exhibit superior effectiveness in mitigating positive symptoms, hence creating a significant gap in the management of negative and cognitive symptoms of schizophrenia **(**Moya et al. 2023). The neurobiological basis of schizophrenia is established through a complex interaction between genetic, neurochemical, structural, and neurodevelopmental variables. Genetic research identified many loci linked to schizophrenia risk, highlighting the disorder’s polygenic characteristics **(**Schmitt et al. 2024).

The precise etiology of schizophrenia is poorly understood. Two prominent theories concerning the neurological basis of the disease focus on the roles of dopamine and glutamate **(**Howes et al. 2015). The dopamine hypothesis of schizophrenia initially proposed that psychosis is associated with heightened dopamine activity **(**Moghaddam and Homayoun 2007**)**. Studies indicate that while dopamine plays a role in the pathophysiology of schizophrenia, its dysfunction does not fully explain all aspects of the disorder, particularly the underlying pathology of negative or cognitive symptoms, underscoring the necessity for a deeper understanding of alterations in other systems **(**Abi-Dargham and Meyer 2014**)**. The N-methyl-D-aspartate receptor (NMDAR) hypofunction theory of schizophrenia suggests that disruptions in the glutamatergic system, namely NMDAR impairment, are fundamental to the disorder’s etiology **(**Zhand et al. 2019). This theory is supported by evidence indicating that NMDAR antagonists, such as phencyclidine (PCP) and ketamine, can elicit symptoms similar to schizophrenia, including positive, negative, and cognitive deficits **(**Hu et al. 2015; Nakazawa and Sapkota 2020**)**. Hence, the glutamate hypothesis accounts for both positive and negative symptoms, as well as cognitive impairments, in contrast to the dopamine hypothesis **(**Mei et al. 2018). However, neurotransmitter-centric theories do not explain all aspects of schizophrenia pathology, prompting investigation into other mechanisms such as protein misfolding and cellular stress responses.

Proteinopathy, also known as protein conformational disorder, refers to a category of disorders characterized by the misfolding of proteins, resulting in cellular, tissue, and organ dysfunction **(**Dugger and Dickson 2017**)**. These misfolded protein aggregates exhibit resistance to clearance and can disrupt the normal functioning of the affected organs. Furthermore, misfolding may result in the loss of the protein’s typical function **(**Ajmal 2023**)**. Growing evidence indicates that proteostasis disturbances and consequently the buildup of misfolded or aggregated proteins in the brain may be implicated not only in neurodegenerative illnesses but also in mental disorders such as schizophrenia, bipolar disorder, and depression **(**Ochneva et al. 2022).

The endoplasmic reticulum (ER) is crucial for protein synthesis, folding, and quality control, maintaining correct protein homeostasis **(**Araki and Nagata 2011**)**. Accumulation of misfolded proteins induces ER stress, triggering the unfolded protein response (UPR) to reestablish homeostasis. Prolonged ER stress can result in neuroinflammation, oxidative stress, and cellular apoptosis which are the main pathological events associated with schizophrenia **(**Cao and Kaufman 2014**)**. Research indicates that ER stress is also implicated in synaptic dysfunction, neurotransmitter imbalances, and cognitive impairments **(**Patel et al. 2017).

The sigma-1 receptor (Sig-1R) is an ER-resident chaperone protein essential for modulating ER stress, sustaining Ca^2+^ homeostasis, and providing neuroprotection **(**Botond et al. 2018). Sig-1R, prominently concentrated in the central nervous system (CNS), participates in various cellular processes, such as Ca^2+^ signaling, protein quality control, neuritogenesis, microglial activation, and ion channel regulation **(**Tsai et al. 2014; Aishwarya et al. 2021; Shi et al. 2021). Sig-1R functions as a pro-survival and anti-apoptotic protein, especially during extended ER stress, where it alleviates cellular damage and enhances resilience **(**Ryskamp et al. 2019). Impairment or reduced functionality of Sig-1R has been associated with cognitive deficits, neuroinflammation, and mitochondrial abnormalities **(**Maurice and Goguadze 2017**)**. Thus, the regulation of Sig-1R is under investigation as a potential therapeutic approach to alleviate ER stress, improve neuronal health, and reduce symptoms of schizophrenia **(**Tsai et al. 2014).

The aim of this review is to present the significance of protein misfolding in schizophrenia, emphasizing ER stress and the involvement of Sig-1Rs in the disease’s progression. Additionally, this review indicates the impact of ER stress and UPR dysregulation on synaptic dysfunction, neuroinflammation, and neuronal death, while highlighting the protective role of Sig-1R in regulating ER stress, Ca^2+^ signaling, and neurotransmitter balance. Further, the viability of targeting Sig-1R as a therapeutic approach to enhance proteostasis, alleviate neuronal dysfunction, and improve clinical outcomes in schizophrenia is assessed herein.

## Common pathways of protein misfolding and aggregation

The formation of protein aggregates in cells arises from the disruption of various physiological processes, including ER stress, chaperone protein deficiencies, and mitochondrial malfunction. These disturbances either result in the creation and aggregation of misfolded proteins within the cell or obstruct the usual mechanisms responsible for the removal of protein aggregates.

### Disruption to the ER under stress

The ER is central to protein synthesis, folding, and post-translational modifications in eukaryotic cells (Almanza et al. 2019). Proteostasis denotes the dynamic coordination necessary for accurate protein folding, quality assurance, and degradation. Chaperone proteins are essential in this process by identifying non-native protein conformations and facilitating the correct re-folding (Díaz-Villanueva et al. 2015). The ER maintains proteostasis through interactions with mitochondria at mitochondria-associated membranes (MAMs), which constitutes approximately 20% of ER-mitochondrial contact sites and regulate Ca^2+^, reactive oxygen species (ROS), lipid metabolism, inflammation and apoptosis (Theurey and Rieusset 2017; Sukhorukov et al. 2022). When folding capacity is exceeded, ER stress arises, leading to unfolded protein accumulation. Stressors such as hypoxia, malnutrition, or Ca^2+^ imbalance activate the UPR, a signaling network aimed at restoring ER homeostasis by reducing translation, enhancing folding, and promoting clearance of misfolded proteins through ER-associated degradation (ERAD) and autophagy (Walter and Ron 2011; He et al. 2024). If unresolved UPR signaling shifts towards apoptosis (Shore et al. 2011). The UPR is mediated by three ER transmembrane sensors, PERK, IRE1, and ATF6. Under physiological conditions, these sensors are suppressed by the interaction of their luminal domains with BiP/GRP78 (78-kDa glucose-regulated protein), the predominant chaperone residing in the ER. Accumulation of unfolded proteins causes BiP dissociation, activating these pathways (Pincus et al. 2010; Oakes and Papa 2015**)**. Upon activation, PERK phosphorylates eukaryotic initiation factor 2α (eIF2α), diminishing its activity and decelerating global protein translation, while promoting activating transcription factor 4 (ATF4) expression, which regulates genes associated with antioxidant responses as well as amino acid metabolism and transport, hence enhancing cellular longevity (Ameri and Harris 2008; Donnelly et al. 2013; Pakos-Zebrucka et al. 2016). Moreover, ATF4 upregulates C/EBP homologous protein (CHOP), which subsequently induces the transcription of DNA damage-inducible protein 34 (GADD34) to restore protein synthesis by dephosphorylating eIF2α (Choy et al., 2015). Excessive CHOP activation, however, suppresses the anti-apoptotic protein B-cell lymphoma 2 (Bcl-2) and enhances pro-apoptotic Bcl-2 interacting mediator (BIM) expression, thereby promoting mitochondria-dependent apoptosis (Rozpedek et al. 2016). IRE1 activation induces splicing of X-box-binding protein 1 (XBP1) mRNA, producing XBP1s, which is associated with protein folding, trafficking, the ERAD pathway, and lipid biosynthesis (Siwecka et al. 2021; Chen et al. 2023). However, under chronic stress, IRE1 promotes apoptosis via TNF receptor-associated factor 2 (TRAF2)-apoptosis signal-regulating kinase 1 (ASK1)-c-Jun N-terminal kinase (JNK) and caspase-12 activation as well as degrading ER mRNAs via regulated IRE1-dependent decay (RIDD) (Hollien and Weissman 2006). ATF6 translocates to the Golgi for activation, inducing chaperones and XBP1, and under prolonged stress, also contributes to CHOP-mediated apoptosis (Bueter et al. 2009; Simoni et al. 2022).

### Defects in chaperone proteins

Chaperone proteins are essential for ER function, facilitating the correct folding of polypeptides and inhibiting protein misfolding and aggregation. They additionally facilitate the synthesis of functional proteins and their transport from the ER **(**Hendershot et al. 2024). This mechanism can be impeded by pathological circumstances including oxidative stress, inflammation, and variations in Ca^2+^ concentrations. These disruptions result in the aggregation of misfolded proteins within the ER lumen **(**Ochneva et al. 2022). ER chaperones, including heat shock protein 5 (HSPA5), often referred to as the 78 kDa glucose-regulated protein (GRP78), are essential for refolding the misfolded proteins to reestablish ER’s homeostasis after stress **(**Sakurai et al. 2007).

#### Sigma-1 receptors (sig-1Rs)

**Fig. 1:**
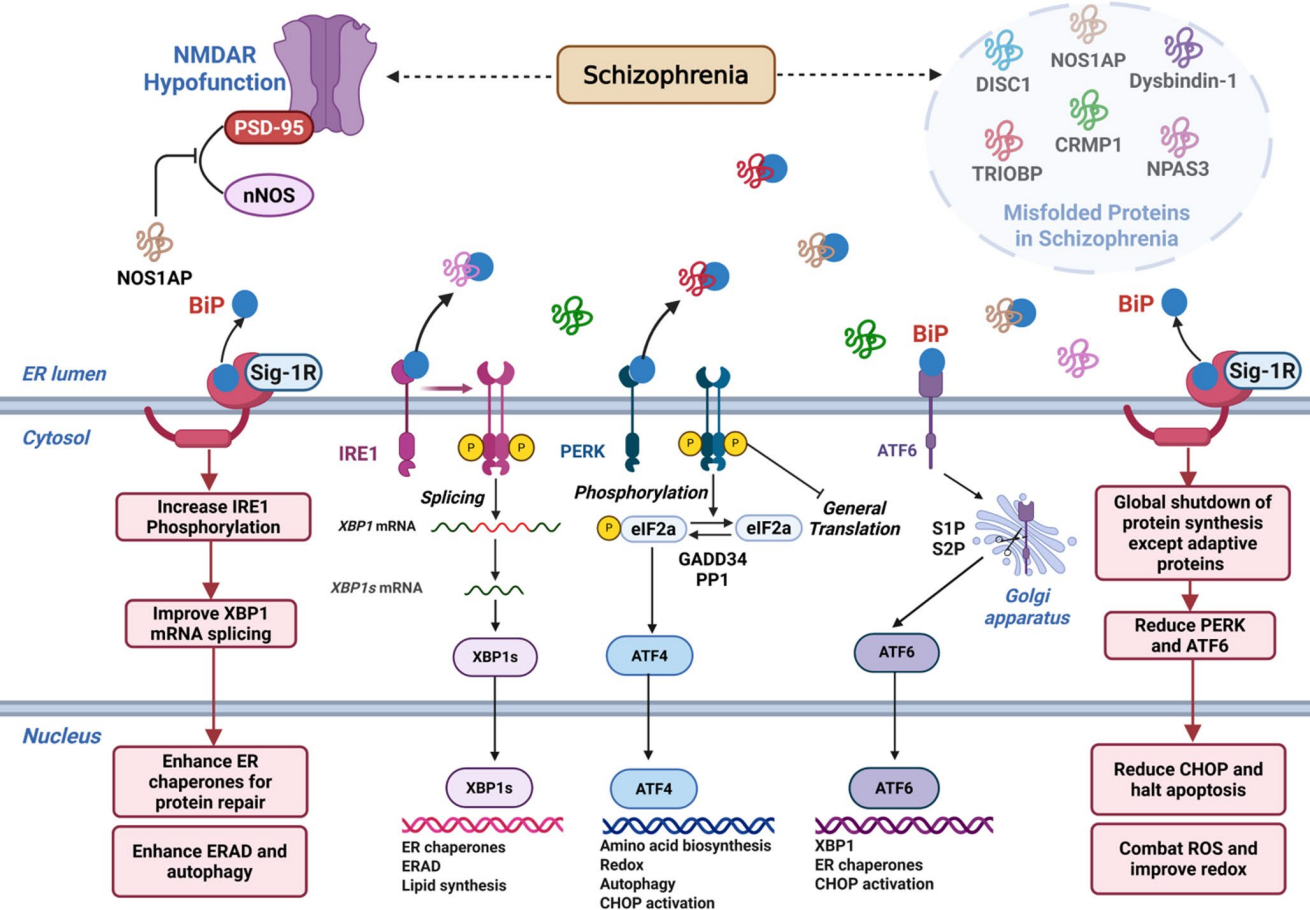
Systematic overview of misfolded proteins and NMDA receptor hypofunction in schizophrenia, highlighting ER stress and sigma-1 receptor functions. This figure illustrates the interplay between NMDAR hypofunction, protein misfolding, and ER stress in schizophrenia, emphasizing the regulatory role of Sig-1R. NMDAR hypofunction, through disrupted interactions between PSD-95 and nNOS, contributes to synaptic dysfunction and increased vulnerability to ER stress. Misfolded proteins commonly associated with schizophrenia, including DISC1, CRMP1, dysbindin-1, NOS1AP, NPAS3, and TRIOBP, accumulate within the ER, activating unfolded protein response (UPR) pathways mediated by IRE1, PERK, and ATF6. Activation of Sig-1R enhances IRE1 phosphorylation and XBP1 mRNA splicing, promoting ER chaperone expression, autophagy, and protein repair. Concurrently, Sig-1R suppresses excessive PERK and ATF6 signaling, reducing CHOP-mediated apoptosis and oxidative stress. Collectively, these mechanisms highlight how Sig-1R functions as a protective chaperone, maintaining ER and neuronal homeostasis against schizophrenia-related proteostatic and synaptic disturbances. NMDAR: N-methyl D-aspartate receptor, Sig-1R: sigma-1 receptor, ER: endoplasmic reticulum, PSD-95: postsynaptic density protein-95, nNOS: neuronal nitric oxide synthase, DISC1: disrupted-in-Schizophrenia 1, CRMP1: collapsin response mediator protein 1, NOS1AP: nNOS adaptor protein, NPAS3: neuronal PAS domain protein, TRIOBP: TRIO and F-actin binding protein, UPR: unfolded protein response, IRE1: inositol-requiring enzyme type 1, PERK: protein kinase R-like ER kinase, ATF6: activating transcription factor 6, XBP1: X-box-binding protein 1, CHOP: C/EBP homologous protein

**Fig. 2:**
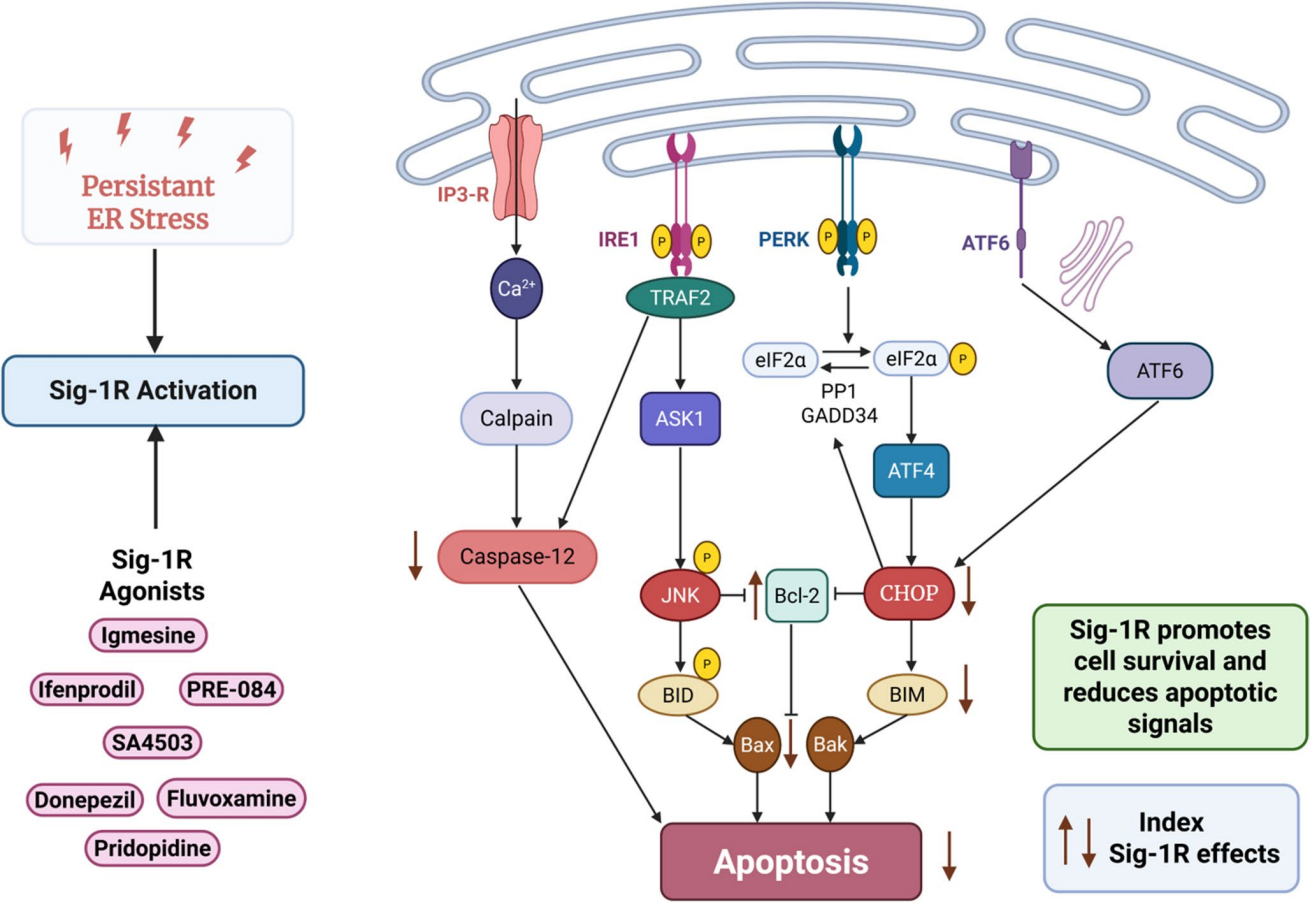
Systematic overview of Sig-1R modulation of ER stress-induced apoptosis. This figure illustrates how activation of Sig-1R mitigates persistent ER stress and prevents apoptotic signaling. Under ER stress conditions, Sig-1R activation, either through endogenous mechanisms or Sig-1R agonists such as igmesine, ifenprodil, PRE-084, SA4503, donepezil, fluvoxamine, and pridopidine, enhances cellular adaptation and survival. Sig-1R suppresses ER stress sensors (IRE1, PERK, and ATF6) that mediate pro-apoptotic pathways, leading to reduced activation of CHOP and caspase-12, while upregulating the anti-apoptotic protein Bcl-2. Consequently, Sig-1R activation inhibits Bax/Bak-mediated apoptosis, restores ER homeostasis, and promotes neuronal survival under conditions of chronic ER stress. CHOP: C/EBP homologous protein, ER: endoplasmic reticulum, IRE1: inositol-requiring enzyme type 1, PERK: protein kinase R-like ER kinase, ATF6: activating transcription factor 6, Bax: bcl-2- associated X protein, BIM: Bcl-2 interacting mediator, BID: BH3-interacting domain death agonist, Sig-1R: sigma-1 receptor, ASK1: apoptosis signal-regulating kinase 1

Sig-1Rs are molecular chaperones regulated by ligands, mostly situated in the ER, particularly at MAM Hayashi and Su 2007; Hayashi 2015; Schmidt et al. 2016). However, they are not limited to the MAM; upon ligand activation, they can relocate to other cellular or neuronal areas, where they engage with and influence the function of diverse receptors, ion channels, and kinases on the plasma membrane (Shi et al. 2021). Under physiological conditions, Sig-1R remains bound to the ER- resident chaperone BiP/GRP78 maintaining it in an inactive state. During conditions of ER stress or accumulation of misfolded proteins, Sig-1R dissociates from BiP and becomes functionally active (Hayashi and Su 2007). Once activated, either by stress signals or pharmacological agonists, Sig-1R exerts several coordinated functions to restore protein homeostasis (Mori et al. 2012; Tsai et al. 2014) (Fig. 1). First, by stabilizing the inositol 1,4,5-trisphosphate type 3 (IP3) receptors, Sig-1R ensures balanced calcium transfer to mitochondria, sustaining ATP production and preventing calcium-induced stress that can lead to protein misfolding (Rodríguez et al. 2022). It also supports the proper folding of nascent proteins and enhances cellular resilience to oxidative damage by fine-tuning ROS generation and activating anti-oxidant defenses (Pal et al. 2012; Eskandari et al. 2025). Second, Sig-1Rs plays a pivotal role in modulating ER stress and UPR to maintain homeostasis (Su et al. 2010; Mori et al. 2013). Upon activation, Sig-1R interacts with ER stress sensors IRE1, PERK, and ATF6 to fine-tune adaptive signaling pathways. It enhances IER1-mediated splicing of XBP1 mRNA, which promotes the transcription of ERAD components, molecular chaperones and lipid biosynthetic enzymes that assist in protein folding and clearance (Mori et al. 2012; Alam et al. 2017). Simultaneously, Sig-1R suppresses the expression of the pro-apoptotic factor CHOP and preserves mitochondrial integrity by maintaining BCL-2 family balance, thereby mitigating cell death under prolonged stress conditions (Zhao et al. 2019). Moreover, by regulating the PERK pathway and eIF2α phosphorylation, Sig1R helps reducing global protein synthesis while selectively enhancing the translation of stress-adaptive genes such as ATF4 (Eskandari et al. 2025). Also, upon stress induction, Sig-1R expression is upregulated via the PERK/eIF2α/ATF4 pathway, and its overexpression suppresses the excessive activation of key UPR stressors, such as PERK and ATF6, further reducing the expression of CHOP and inhibiting apoptosis (Mitsuda et al. 2011) (Fig. 2). Third, Sig-1R facilitates the removal of irreversibly misfolded proteins by regulating autophagy pathways. It interacts with autophagy regulators such as Beclin-1 and microtubule-associated protein light chain 3 (LC3), promoting autophagosome formation and lysosomal clearance of aggregated proteins (Vollrath et al. 2014; Christ et al. 2019; Knupp et al. 2024; Eskandari et al. 2025). Finally, Sig-1R suppresses microglial pro-inflammatory signaling and TNF-α release, reducing inflammation induced by misfolded proteins and preserving neuronal integrity (Hall et al. 2009; Jia et al. 2018). These combined actions make Sig-1R a key regulator of proteostasis, linking ER chaperone function, UPR modulation, and autophagy regulation mechanisms, that are particularly relevant to neurodegenerative and psychiatric disorders.

## Mechanisms of protein misfolding and aggregation in schizophrenia

Taken together, the ER-stress and chaperone-dysfunction mechanisms outlined above appear to converge on neuronal circuits that are disrupted in schizophrenia, and accumulating post-mortem, genetic, and transcriptomic evidence now implicates chronic UPR activation and insufficient proteostatic buffering as direct drivers of the disorder’s synaptic and cognitive pathology.

Schizophrenia is linked to abnormalities in all physiological mechanisms of protein aggregation, including ER stress and deficiencies in chaperone proteins. Moreover, some psychiatric drugs may incidentally modulate ER stress responses, though this is not their primary mechanism of action. The suggested sequence of events in the development of mental disorders involves the escalation of severe or chronic ER stress, accompanied by elevated levels of ROS and Ca^2+^, leading to the upregulation of transcription factors (CHOP) and kinases (JNK) through the activation of the pro-apoptotic pathway of the UPR. The failure of the UPR results in the accumulation of protein aggregates. This accumulation activates the inflammasome, leading to increased inflammatory signaling through pro-inflammatory cytokines. Subsequently, pro-apoptotic caspases are triggered, resulting in increased neuronal death.

### Dysregulation of the unfolded protein response and chaperone proteins in schizophrenia

Studies have progressively examined protein misfolding and its association with the UPR and chaperone proteins within the ER in the context of schizophrenia **(**Srinivasan et al. 2020; Kim et al. 2021yükada et al., 2023). Kim et al. (2021) observed an increase in BiP expression, a drop in PERK levels, and a reduction in IRE1 phosphorylation. This study identified elevated levels of XBP1 protein and spliced XBP1 mRNA in the dorsolateral prefrontal cortex of elderly patients with schizophrenia **(**Kim et al. 2021). XBP1 polymorphism was associated with schizophrenia in the Japanese population; however, these findings were not consolidated by subsequent research **(**Watanabe et al. 2006). Another study identified aberrant expression of UPR-related genes in the prefrontal cortex of individuals with schizophrenia. Serum levels of ATF6 and XBP1 were shown to be elevated in patients with schizophrenia, suggesting a potential systemic dysregulation of the unfolded protein response in this disorder **(**Xue et al. 2023). Furthermore, researchers discovered evidence of an interaction between disrupted-in-schizophrenia 1 (DISC-1), a protein susceptible to aggregation, and ATF4 within the PERK pathway **(**Trinh et al. 2012; Ochneva et al. 2022). Trinh et al. (2012) discovered that the levels of PERK and ATF4 were significantly diminished in the frontal cortex of schizophrenia patients relative to the control group. Also, many studies emphasized PERK’s involvement in cognitive impairment in rodents. The prefrontal cortex of PERK-deficient animals exhibited reduced eIF2α phosphorylation and ATF4 expression, resulting in heightened behavioral perseveration and diminished behavioral flexibility **(**Trinh et al. 2012; Srinivasan et al. 2020).

Research indicates that chaperone protein pathology contributes to psychiatric disorders. Sig-1Rs were identified as being associated with the etiology of various neurological disorders, including schizophrenia **(**Ishikawa and Hashimoto 2009; Takizawa et al. 2009). A notable reduction in Sig-1Rs was detected in the brains of individuals with schizophrenia **(**Takizawa et al. 2009; Mitsuda et al. 2011). Sig-1R knockout mice exhibited significant cognitive impairments, including deficits in memory and learning **(**Maurice et al. 2019). Furthermore, the lack of Sig1Rs in motor neurons induces ER stress, which impacts mitochondrial dynamics and functionality **(**Watanabe et al. 2016; Sukhorukov et al. 2022). As a result, the modulation of the chaperone system has lately become significant as a prospective therapeutic approach for disorders associated with defective protein folding **(**Suzuki 2014**)**. This method may entail modifying chaperone gene expression or employing compounds that augment chaperone activity **(**Omi et al. 2014; Taldone et al. 2014). These techniques are presently under investigation, especially for the treatment of neurodegenerative diseases and other disorders linked to protein misfolding **(**Tomihisa et al. 2012; Wang et al. 2021; Sharma et al. 2024).

### Autophagy dysfunction and schizophrenia

Autophagy is an evolutionarily conserved cellular quality-control mechanism that ensures the clearance of misfolded proteins, damaged organelles, and surplus cellular components to maintain proteostasis and neuronal survival. The process involves the initiation of a double-membrane phagophore, which matures into an autophagosome that subsequently fuses with a lysosome for the degradation of its cargo **(**Klionsky 2005**)**. This tightly regulated process depends on autophagy-related genes (ATGs) and is controlled by key signaling pathways such as the mammalian target of rapamycin (mTOR) and AMP-activated protein kinase (AMPK), which regulate unc-51-like kinases 1 and 2 (ULK1/2), Beclin-1, and other components of the core autophagy machinery **(**Chan 2012; Lamb et al. 2013; Cao et al. 2016). Dysfunction of the system disrupts neuronal homeostasis, leading to the accumulation of toxic proteins and damaged organelles that precipitate in synaptic dysfunction and neuronal loss **(**Morimoto and Cuervo 2009**)**. In the context of schizophrenia, increasing evidence indicates that autophagy is impaired where postmortem analyses of patient brain tissue reveal reduced expression of ATG such as Beclin-1, ULK2, and ATG3, particularly in Brodmann area 22, a region implicated in positive symptoms such as hallucinations. Such findings support the hypothesis that defective autophagy may contribute to schizophrenia pathology **(**Kroemer et al. 2010; Horesh et al. 2011). Sig-1R has emerged as a critical regulator of autophagy and proteostasis **(**Prasanth et al. 2021; Prasansuklab et al. 2025). Experimental studies demonstrate that Sig-1R agonists such as PRE-084 and ANAVEX2-73 induce autophagosome biogenesis, partly via enhanced ULK1 phosphorylation, while additional evidence confirms that autophagosomes are formed at MAM regions where Sig-1Rs are concentrated **(**Hamasaki et al. 2013; Christ et al. 2019). Conversely, genetic knockout (KO) of Sig-1R impairs autophagosome clearance, whereas transfection with full-length, but not truncated, Sig-1R restores the fusion of phagophores with lysosomes, confirming its essential role in autophagic regulation **(**Mizushima and Levine 2010; Yang et al. 2019). Beyond these models, Sig-1R ligands exhibit differential effects on autophagic flux depending on their agonist or antagonist profile, with several clinically used drugs, including the Sig-1R agonists fluoxetine and fluvoxamine, also shown to promote autophagy and increase clearance of damaged mitochondria **(**Li et al. 2017). Importantly given its dual role as ER chaperone and regulator of protein degradation, Sig-1R provides a mechanistic link between autophagy and misfolded protein clearance. This convergence is particularly relevant to schizophrenia, where autophagy dysfunction and impaired proteostasis are increasingly implicated in disease pathophysiology. Thus, pharmacological targeting of Sig-1R with agonists or allosteric modulators offers a promising therapeutic strategy to restore autophagic balance, enhance clearance of toxic proteins, and alleviate cellular stress underlying schizophrenia **(**Yang and Xu 2020; Prasanth et al. 2021).

### Misfolded and aggregated proteins in schizophrenia

Schizophrenia is increasingly associated with protein homeostasis disruptions such as misfolding, aggregation, and impaired degradation, which collectively impair synaptic function, neurotransmission, and neuronal signaling, resulting in cognitive deficits, neuroinflammation, and neurodegeneration **(**Ochneva et al. 2022). These anomalies could be caused by genetic mutations, oxidative damage, ER stress, or failure in important protein degradation mechanisms such as the ubiquitin-proteasome system (UPS) and autophagy **(**Li et al. 2022). In addition, dysregulation of molecular chaperones and heat shock proteins (HSPs) interferes with correct protein folding, worsening cellular stress and neuronal dysfunction. Misfolded proteins can accumulate and in turn activate neuroinflammatory pathways, resulting in prolonged glial activation and the release of pro-inflammatory cytokines that damage neuronal integrity **(**Brezic et al. 2025). Moreover, deficiencies in the UPR compromise protective mechanisms designed to restore proteostasis, increasing susceptibility to apoptosis and neurodegeneration. Misfolded proteins like disrupted-in-Schizophrenia 1 (DISC1), dysbindin, and neuronal nitric oxide synthase 1 adaptor protein (NOS1AP) disrupt intracellular signaling and synaptic plasticity, contributing to schizophrenia’s structural and functional brain abnormalities, especially in cognition-related regions like the prefrontal cortex and hippocampus **(**Brzustowicz 2008; Trossbach et al. 2016; Wang et al. 2017). This synaptic dysfunction is hypothesized to underlie the cognitive deficits seen in schizophrenia, which are not improved by dopamine-targeting antipsychotics **(**McCutcheon et al. 2023).

It should be noted, however, that direct evidence of large-scale protein aggregates in schizophrenia brains is not as robust as in classical neurodegenerative diseases, and many findings remain correlational.

#### Disrupted-in-schizophrenia 1 (DISC1)

DISC1 is a protein encoded by the DISC1 gene located on chromosome 1q42.1 **(**Hodgkinson et al. 2004). It is crucial for various neuronal functions, including cell proliferation, differentiation, migration, and synaptic function **(**Korth 2012; Wu et al. 2017). Alterations in DISC1 have been linked to psychiatric illnesses like schizophrenia and bipolar disorder **(**Thomson et al. 2013). Mutations or truncations in the DISC1 gene can lead to the formation of toxic protein aggregates, interfering with important cellular activities. These aggregates disrupt dopamine signaling, cAMP signaling, and cytoskeletal architecture, leading to synaptic dysfunction and neurodevelopmental anomalies in schizophrenia **(**Gamo et al. 2013; Trossbach et al. 2016). Clinical studies have revealed DISC1 mutations in schizophrenia patients, with postmortem examinations showing higher levels of insoluble DISC1 aggregates in their brains **(**Leliveld et al. 2008; Samardžija et al. 2021). Investigations into DISC1 point mutations in mice models of schizophrenia reveal that protein aggregation can result from increased intermolecular cohesion, causing brain dysfunction. Also, a decrease in total or functional DISC1disrupts sensorimotor gating, a prevelant hallmark of schizophrenia **(**Kvajo et al. 2008; Kakuda et al. 2018). Transgenic rat models demonstrate that DISC1 has a role in postnatal neurogenesis and, alongside environmental variables, contributes to the dysregulation of the dopamine system **(**Inta et al. 2010). Moreover, it was reported that the overexpression of full-length DISC1 in rats led to aggregates formation with subsequent precipitation of schizophrenia-like molecular and behavioral abnormalities **(**Trossbach et al. 2016).

#### Neuronal nitric oxide synthase 1 adaptor protein (NOS1AP)

NOS1AP, referred to as CAPON, is a cytosolic protein that engages with neuronal nitric oxide synthase (nNOS), profoundly affecting nitric oxide (NO) signaling, synaptic plasticity, and neuronal development. It function as an adaptor protein that enhances signaling within a complex of the NMDA receptor, PSD-95, and nNOS, therefore modulating neuronal NO production **(**Matiiv et al. 2022). The NMDAR-PSD95-nNOS triplet complex is essential for various neuronal activities, including synaptic plasticity, cellular survival, and cognitive abilities such as learning and memory **(**Maccallini and Amoroso 2016; Zhang et al. 2018). CAPON can compete with PSD-95 for binding to nNOS, disrupting the assembly of the NMDAR-PSD-95-nNOS complex and facilitating the development of a novel complex, NMDAR-CAPON-nNOS **(**Jaffrey et al. 1998). This alternative complex diminishes nNOS activity, hence decreasing NO production, which is essential for maintaining neuronal metabolism and function by preventing excessive nNOS activation. However, the overexpression of CAPON may significantly hinder the appropriate assembly of NMDAR-PSD95-nNOS, potentially resulting in neuronal malfunction **(**Nikonenko et al. 2008). The association between NOS1AP and schizophrenia has been emphasized by genetic and biochemical studies, demonstrating its significance in disease pathogenesis. Bioinformatics analysis has identified NOS1AP as a protein prone to aggregation that interacts with α-synuclein, a critical component in neurodegenerative diseases. Overproduction of NOS1AP results in the formation of detergent-resistant, non-amyloid aggregates, which may potentially contribute to neurodegenerative and mental disorders **(**Matiiv et al. 2022). Post-mortem brain tissues from the dorsolateral prefrontal cortex of individuals with schizophrenia have shown elevated NOS1AP expression **(**Brzustowicz 2008**)**. Furthermore, elevated levels of NOS1AP mRNA and protein have been observed in both the blood and dorsolateral prefrontal cortex of patients with schizophrenia **(**Xu et al. 2005; Hadzimichalis et al. 2010; Hu et al. 2018). Increased levels of NOS1AP expression have also been noted in rat models of neurological injury **(**Cheng et al. 2008; Cui et al. 2011). Moreover, the overexpression of murine NOS1AP in the dorsal hippocampus of wild-type mice has been associated with impairments in social memory and working memory, behavioral characteristics typically attributed to schizophrenia **(**Freudenberg et al. 2021).

#### Dysbindin-1

Dysbindin-1, encoded by the dystobrevin-binding protein 1 (DTNBP1) gene, is a protein present in cerebral tissue. It is essential for preserving optimal synaptic structure and neurotransmitter balance, particularly within dopamine and glutamate systems **(**Jun et al. 2022). Dysbindin is a constituent of the lysosome-1-associated organelle biogenesis complex (BLOC-1) within the synapse, modulates NMDA and D2 receptors on the membrane surface, and participates in vesicular transport **(**Yang et al. 2016b). The human DTNBP1 gene undergoes alternative splicing, yielding a minimum of three distinct isoforms of the dysbindin protein: dysbindin-1 A, −1B, and − 1 C **(**Xu et al. 2015). These aggregates exhibit neurotoxicity and possess the ability to disseminate through exosomal transport. Moreover, dysbindin-1 A interacts with dysbindin-1B and is recruited to the aggresome structure when co-expressed with dysbindin-1B (Zhu, et al., 2015). Research with transgenic mice showed that the overexpression of dysbindin-1B can result in its aggregation and coaggregation with BLOC-1 subunits, hence disrupting normal BLOC-1 function, and thereby impairing synaptic vesicle trafficking **(**Yang et al. 2016b). A study by **(**Shintani et al. 2014) examined transgenic mice that overexpressed human dysbindin-1 and discovered that these mice displayed deficits in working memory and social interactions, behavioral characteristics linked to schizophrenia. Research has discovered increased mRNA levels of genes encoding dysbindin-1B in people diagnosed with schizophrenia **(**Shintani et al. 2014). Moreover, particular intronic mutations in the DTNBP1 gene, linked to schizophrenia, have been demonstrated to affect alternative splicing, leading to elevated expression of dysbindin-1B **(**Xu et al. 2015).

#### Collapsin response mediator protein 1 (CRMP1)

CRMPs are crucial phosphoproteins that exhibit high expression throughout brain development, consisting of five homologous cytosolic proteins (CRMP1-5) **(**Lin et al. 2019). The CRMP family proteins are distinguished by their classification as a novel group of microtubule-associated proteins that are integral to various processes in nervous system development, such as axon guidance, synapse maturation, cell migration, and even in adult brain function, including axon guidance, synapse maturation, cell migration, as well as adult brain functions **(**Khazaei et al. 2014; Nakamura et al. 2020). In a recent study, Bader and colleagues identified CRMP1 as the epitope for an antibody that differentiate between a collection of aggregates obtained from brain samples of schizophrenia patients and a comparable collection from control subjects **(**Bader et al. 2012b). CRMP1 has been identified to exist in two splice variants: the short variant (sv) and the long variant (lv). While the short isoform of CRMP1 does not aggregate independently; instead, its co-expression with DISC1 results in co-aggregation, producing insoluble precipitates in conjunction with DISC1 aggregates. This relationship indicates that CRMP1 may be integral to the pathogenic processes of schizophrenia through its functional interaction and intersection of DISC1-related pathways **(**Bader et al. 2012a, b).

#### TRIO and f-actin binding protein (TRIOBP)

The TRIOBP gene encodes several proteins that are crucial for regulating the formation of the actin cytoskeleton. The TRIOBP gene undergoes complex alternative splicing, yielding many protein variants, such as TRIOBP-1 and TRIOBP-4. TRIOBP-1 is a mostly structured protein that is universally expressed and interacts with F-actin, inhibiting its depolymerization. It has been demonstrated to be crucial for various activities, including the cell cycle, adhesion junctions, and neural development. TRIOBP-1 has been associated with schizophrenia via the production of protein aggregates in the brain **(**Zaharija et al. 2020). An antibody targeting TRIOBP-1 exhibited specificity for pooled pure insoluble protein fractions derived from post-mortem brain samples of schizophrenia patients, in contrast to a comparable preparation from brain samples of healthy individuals **(**Bradshaw et al. 2017). Despite limited research on its brain-specific functions, TRIOBP-1 remains a crucial regulator of actin polymerization and is involved in chromosome segregation and cell migration, interacting with nuclear distribution element-like 1 (NDEL1), a neurodevelopmentally significant protein associated with schizophrenia **(**Seipel et al. 2001; Hong et al. 2016; Bradshaw and Hayashi 2017**)**. Postmortem investigations have demonstrated modest yet significant elevations in TRIOBP transcript expression among individuals with schizophrenia **(**Maycox et al. 2009).

#### Neuronal PAS domain protein 3 (NPAS3)

NPAS3 encodes a member of the basic helix-loop-helix (bHLH) PAS domain transcription factor family. These proteins are essential for neurogenesis, metabolic processes, and the regulation of circadian rhythms **(**Yang et al. 2016a). The disruption of the NPAS3 gene in a Scottish mother and daughter diagnosed with schizophrenia and minor learning difficulties offered the initial evidence of this gene’s involvement in mental disorders **(**Kamnasaran et al. 2003; Pickard et al. 2005). NPAS3 is inherently prone to aggregation, a tendency that is intensified by the valine to isoleucine (V304I) amino acid substitution mutation. This mutation has been demonstrated to modify transcriptional activity, reduce soluble endogenous NPAS3, and elevate its insoluble portion, suggesting a destabilizing impact on the protein’s functional domain **(**Nucifora et al. 2016). A recent study examined NPAS3 aggregation with a purified insoluble fraction of human insular cortex homogenates, revealing full-length NPAS3 in 70% of the specimens. These data indicate that NPAS3 aggregation is quite prevalent and not exclusively limited to the V304I mutation **(**Samardžija et al. 2021).

Collectively, these findings illustrate that a diverse set of proteins (DISC1, NOS1AP, dysbindin, etc.) show abnormal aggregation or misfolding in schizophrenia, potentially converging on common downstream effects such as impaired synaptic plasticity, disrupted intracellular signaling, and activation of stress responses (e.g. ER stress and inflammation). Sig-1Rs, as molecular chaperones, contribute in maintaining proteostasis by stabilizing client proteins, facilitating the refolding of misfolded proteins, and supporting adaptive quality-control pathways, such as the UPR and autophagy. By engaging into these protein repair mechanisms, Sig-1Rs protect against toxic protein accumulation and promotes neuronal survival.

Finally, we summarized the data on the proteins that could potentially be involved in misfolding and aggregation processes in schizophrenia **(**Table 1**)**.

**Table 1:** Misfolded and aggregated proteins in schizophrenia

Protein	Functions	Species	References
DISC1	It is a scaffold protein essential for cerebral development and functionality. It facilitates neuronal growth, migration, synaptic transmission, and plasticity, especially via dopamine and glutamate signaling. It is involved in mitochondrial function, intracellular transport, and adult neurogenesis.	Human	**(**Leliveld et al. 2008; Samardžija et al. 2021)
		Rats	**(**Trossbach et al. 2016)
NOS1AP	It is a cytosolic adaptor protein that modulates neuronal signaling through its interaction with neuronal nNOS. This interaction regulates the synthesis of NO, essential for synaptic plasticity, learning, and memory. It additionally affects intracellular signaling, cytoskeletal dynamics, and neuronal growth, morphology, and migration throughout brain development.	Human	**(**Xu et al. 2005; Brzustowicz 2008; Hadzimichalis et al. 2010; Hu et al. 2018)
		Mice	**(**Freudenberg et al. 2021)
Dysbindin-1	It plays an essential role in synaptic function and neuronal communication. It regulates neurotransmitter release, especially glutamate, and plays a role in synaptic plasticity, crucial for learning and memory. Moreover, it is involved in dendritic spine morphology and the formation of neural circuits.	Human	**(**Xu et al. 2015)
		Mice	**(**Shintani et al. 2014)
CRMP1	It is a cytosolic phosphoprotein that is essential for neuronal development and function. It participates in axonal guidance, dendritic spine development, and neuronal polarity, chiefly by regulating microtubule dynamics. It facilitates signaling pathways triggered by semaphorins, which are crucial for axon repulsion and the formation of neural circuits during development. Besides its function in neurodevelopment, CRMP1 also plays a role in synaptic plasticity and is associated with learning and memory processes.	Human	**(**Bader et al. 2012b)
TRIOBP	It is a cytoskeletal protein crucial for the stabilization and organization of actin filaments, which maintain cell morphology and facilitate intracellular transport. It modulates neurite outgrowth, synaptic architecture, and cellular migration in neurons throughout brain development. It facilitates F-actin bundling and preserves cytoskeletal integrity in addition to its role in mitotic spindle assembly and chromosome segregation.	Human	**(**Maycox et al. 2009; Bradshaw et al. 2017)
NPAS3	It is a transcription factor specific to the brain, crucial for neurodevelopment, neuronal differentiation, and synaptic plasticity. It modulates gene expression in reaction to environmental stimuli by forming heterodimers with other transcription factors. It regulates neurogenesis, circadian rhythms, and metabolic pathways as well as hippocampal development and cognitive functions, such as learning and memory.	Human	**(**Samardžija et al. 2021)

## Therapeutic approaches

It is essential to identify and develop specific therapeutic interventions that target the cellular and molecular pathways underlying proteostasis dysregulation implicated in psychiatric disorders such as schizophrenia. Despite mounting recognition of the significance of protein aggregation, ER stress, and impaired chaperone-mediated protein folding in the neuropathology of these conditions, current therapeutic approaches remain predominantly limited and nonspecific **(**Siwecka et al. 2023). Remarkably, few interventions have been designed to specifically address the protein quality control machinery within the context of psychiatric, rather than classical neurodegenerative, disorders. Mounting evidence suggests that modifying critical components of UPR, enhancing ERAD, and stabilizing chaperone protein networks may alleviate the harmful accumulation of misfolded proteins **(**Halliday and Mallucci 2015; Ghemrawi and Khair 2020**)**. Sig-1R agonists have shown particular promise due to their chaperone-like function at the ER-mitochondria interface, where they regulate calcium homeostasis, oxidative stress, and the accuracy of protein folding **(**Ahmed et al. 2025; Mohamed et al. 2025).

### Sig-1R activation

Sig-1R agonists have attracted interest for their possible therapeutic application in neuropsychiatric disorders through the modulation of neurotransmitter systems, alleviation of cellular stress, and enhancement of neuroprotection **(**Maurice and Su 2009; Albayrak and Hashimoto 2017; Ren et al. 2022b; Rafčíková et al. 2023). The Sig-1R, being an intracellular chaperone protein, is predominantly situated in the ER, where it modulates Ca^2+^ signaling, protein folding, and cellular homeostasis (Nguyen et al. 2015). The dysfunction of Sig-1R has been associated with schizophrenia, and its activation may aid in the restoration of essential neurobiological functions (Tsai et al. 2014). By restoring proteostasis and synaptic health, Sig-1R agonists could therefore ameliorate the negative and cognitive symptoms that remain poorly controlled by current dopamine-centric antipsychotics.

Schizophrenia is associated with NMDAR hypofunction, which has been implicated in the disorder’s etiology, notably with its effects on dopamine signaling pathways. Decreased NMDA receptor activation may result in augmented dopamine release in subcortical areas, including the striatum, hence contributing to positive symptoms such as hallucinations and delusions. Conversely, this hypofunction may lead to diminished dopamine activation in the prefrontal cortex, correlated with negative symptoms and cognitive impairments **(**Collo et al. 2020; Nakazawa and Sapkota 2020**)**. Sig-1R agonists augment NMDAR activity and stabilize dopamine release, thereby enhancing cognitive function. Moreover, these agonists provide neuroprotection by alleviating ER stress, diminishing oxidative damage, and maintaining mitochondrial function, all of which are compromised in schizophrenia **(**Weng et al. 2017). Also, they function as molecular chaperones, stabilizing misfolded and aggregated proteins, regulating Ca^2+^ homeostasis, and inhibiting ER stress-induced apoptosis, a mechanism that leads to neuronal degeneration in schizophrenia. Sig-1R agonists additionally engage with anti-apoptotic pathways to inhibit the overactivation of pro-apoptotic proteins, including caspases and Bax, while enhancing the expression of anti-apoptotic factors such as Bcl-2 **(**Botond et al. 2018; Ahmed et al. 2025). Further, Sig-1R agonists enhance synaptic plasticity by elevating brain-derived neurotrophic factor (BDNF) production, thereby facilitating neuronal survival and connection **(**Chen et al. 2021). They moreover demonstrate anti-inflammatory properties by inhibiting pro-inflammatory cytokines and diminishing microglial activation, perhaps mitigating neuroinflammation associated with schizophrenia **(**Tsai et al. 2014). Table 2 summarizes the multiple effects afforded by Sig-1R agonists in experimental studies.

**Table 2:** Overview of Sigma-1 receptor agonists in preclinical research

Sig-1R Agonists	Pharmacological class	Previous preclinical/clinical studies with Sig-1R mediated effects	References
Fluvoxamine	Selective serotonin reuptake inhibitor (SSRI) primarily used to treat obsessive-compulsive disorder and depression.	- Fluvoxamine reversed PCP-triggered schizophrenia-like symptoms in mice through Sig-1R activation. - Fluvoxamine produced an anti-depressant effect, demonstrated in reduced immobility time in forced swimming test in DBA/2 mice through Sig-1R activation. - Fluvoxamine enhanced nerve growth factor (NGF) induced neurite outgrowth via Sig-1R activation in PC12 cell line. - Fluvoxamine mitigated autistic-like behavior and cerebral pathology in valproic acid induced autism in rats via Sig-1R activation. - Fluvoxamine was associated with a significant improvement in spatial working memory strategy, reflecting enhanced executive function, according to secondary analyses. This finding comes from a randomized, double-blind, placebo-controlled add-on trial (*n* = 48) that evaluated fluvoxamine, titrated up to 150 mg/day over 8 weeks, in patients with chronic schizophrenia.	**(**Hashimoto et al. 2007; Nishimura et al. 2008; Niitsu et al. 2012; Sugimoto et al. 2012; Omi et al. 2014; Albayrak and Hashimoto 2017; Matsushima et al. 2019; Khani and Entezari-Maleki 2022; Uslu et al. 2023; Mohamed et al. 2025)
Donepezil	Acetylcholinesterase inhibitor used to treat Alzheimer’s disease.	- Donepezil exhibited antidepressant-like effects in the forced swimmimg test and improved memory in maze-based learning tasks dizocilpine dizocilpine (MK-801) model of schizophrenia. - When combined with the selective σ₁ receptor agonist PRE-084, donepezil produced enhanced cognitive protection in Alzheimer’s-type memory impairment models.	**(**Csernansky et al. 2005; Maurice et al. 2006; Maurice 2016; Wang and Jia 2023**)**
SA4503 (Cutamesine)	A potent selective Sig-1R agonist with 100x selectivity over Sig-2Rs	- In vivo rat microdialysis showed that SA4503 increases acetylcholine release in the frontal cortex and hippocampus. - In the water-finding (latent learning) task, mice treated with NMDA antagonists like PCP showed impaired learning, reflected by increased latencies to locate water. Administration of SA4503 effectively reversed these deficits, restoring normal performance. - Mice treated with MK-801exhibited significant impairments in spatial working memory (Y-maze) and passive avoidance learning, modeling schizophrenia-like cognitive deficits. SA4503, administered subcutaneously in a dose-dependent range (0.03–1 mg/kg), effectively reversed these impairments.	**(**Kobayashi et al. 1996; Senda et al. 1996; Maurice and Privat 1997; Noda et al. 2001)
Ifenprodil	NMDA receptor antagonist.	- Ifenprodil significantly potentiated NGF-induced neurite outgrowth, in a concentration-dependent manner in PC12 cell line.	**(**Ishima and Hashimoto 2012; Hashimoto 2015**)**
Pridopidine	Dopamine stabilizer (D2 Receptor Partial Agonist/Antagonist at High Doses) used in Huntington’s disease.	- Chronic PCP-treated mice developed memory deficits in novel object recognition. Pridopidine reversed these impairments by Sig-1R activation. - Pridopidine protected mouse primary striatal and cortical neurons as well as human HD iPSC-derived cells from mutant Huntingtin-induced toxicity (nanomolar doses). - In mice with unilateral dopaminergic neuron lesions, daily pridopidine improved motor performance (cylinder, stepping tests) and reversed rotational bias. This was accompanied by nigral neuron preservation, increased dopaminergic fiber density, and elevated striatal BDNF/GDNF and ERK1/2 phosphorylation. - In APP/PS1 transgenic mice, pridopidine promoted synaptogenesis and ameliorated spatial memory impairments. Similarly, in vitro, it protected against glutamate-induced excitotoxicity and oxidative stress via Sig-1R activation.	**(**Sahlholm et al. 2018; Eddings et al. 2019; Francardo et al. 2019; Estévez-Silva et al. 2022)
2-(4-Morpholino) ethyl-1-phenylcyclohexane-1-carboxylate (PRE-084)		- In Albino Swiss and C57BL/6J mice, PRE-084 significantly reduced immobility in the forced swim test, a classic assay for antidepressant screening. - PRE-084 reversed MK-801 (dizocilpine)-induced deficits in tasks such as Y-maze alternation and passive avoidance learning.	**(**Maurice et al. 1994; Skuza and Rogóz 2009; Motawe et al. 2020)
ANAVEX2-73 (blarcamesine)	Dual-action Sig-1R agonist and M₁ receptor modulator.	- ANAVEX2-73 improved motor coordination, balance, sensory responses, hindlimb clasping, and respiratory irregularities in both juvenile and adult mice. - Two-week dosing with ANAVEX2-73 (blarcamesine) in Fragile X (Fmr1 KO) mice normalized hyperactivity in open-field tests, improved associative learning in fear conditioning, and reduced anxiety-like perseveration. These behavioral improvements were accompanied by restoration of BDNF expression in the hippocampus, and PET imaging confirmed dose-dependent Sig-1R occupancy, supporting its Sig-1R-mediated neurotherapeutic action. - Pretreatment with ANAVEX2-73 fully prevented Aβ-induced memory impairments. The drug modulated autophagy, reduced amyloid precursor protein, and improved oxidative stress markers.	**(**Kaufmann et al. 2019; Anavex Life Sciences 2021; Reyes et al. 2021)
Igmesine (JO-1784)		- Igmesine has demonstrated significant cognitive-enhancing effects in preclinical models of aging-related memory impairment. In senescence-accelerated mice (SAMP8), igmesine improved spontaneous alternation, passive avoidance, and place learning in the water maze, indicating enhanced learning abilities.	**(**Maurice et al. 1996; Ren et al. 2022a)

Sig-1R antagonists have been extensively utilized in preclinical models to elucidate the mechanistic pathways mediated by Sig-1R **(**Table 3**)**. By selectively blocking Sig-1Rs activity using compounds such as NE-100, BD 1047 or BD 1063, researchers have been able to demonstrate the receptor’s role in modulating key cellular processes, including Ca^2+^ homeostasis, ER stress response, and neurotransmitter regulation. In models of neuropathic pain, substance use, and psychiatric disorders, Sig-1R antagonists consistently exaggerate pathological behaviors and attenuate biochemical markers, supporting the receptor’s involvement in excitatory neurotransmission and intracellular signaling cascades. The ability to magnify disease-relevant phenotypes through Sig-1R blockade offers compelling evidence that these effects are Sig-1R–dependent, providing a powerful pharmacological tool to map Sig-1R-mediated mechanisms and their potential as therapeutic targets.

**Table 3:** Sigma-1 receptor antagonists explored in preclinical model

Sig-1R Antagonists	Pharmacological class	References
N-(2-(3,4-dichlorophenyl)-N-methyl-2-(dimethylamino) ethylamine (BD 1047)	Dopaminergic (D2 receptors) modulation and selective antagonist of Sig-1R	**(**Sugimoto et al. 2012; Shimazawa et al. 2015)
1-(2-(3,4-dichlorophenyl) ethyl)−4-methylpiperazine (BD 1063)	Highly selective blocker for Sig-1R	**(**Matsumoto et al. 1995; Maurice and Su 2009; Sabino et al. 2009)
4-Methoxy-3-(2-phenylethoxy)- N, N-dipropylbenzeneethaneamine (NE-100)	Selective antagonist of the Sig-1R, with minimal activity at sigma-2 and other receptors.	**(**Hashimoto et al. 2007; Nishimura et al. 2008; Mohamed et al. 2025)
Haloperidol	D2 receptor antagonist and Alpha-1 receptor antagonism	**(**Bai et al. 2017; Milenina et al. 2023)
S1RA (E-52862)	Novel, highly selective, and orally bioavailable Sig-1R antagonist.	**(**Bruna et al. 2018; Vavers et al. 2019)
Spipethiane, and its analogue 2-(1-benzylpiperidin-4-yl) thiochroman-4-one	Spipethiane: A thiochroman derivative that acts as a sigma receptor ligand, with moderate to high affinity for Sig-1Rs and reported to have antagonistic activity at Sig-1Rs in various in vitro and animal models. 2-(1-benzylpiperidin-4-yl) thiochroman-4-one: a structural analogue of spipethiane, designed to enhance binding affinity and selectivity for Sig-1Rs.	**(**Cifani et al. 2020)

### Allosteric modulation of sig-1Rs

Allosteric modulation refers to the modulation of protein function through binding of an effector molecule at a site distinct from the orthosteric or active site. This interaction induces conformational changes in the protein, thereby altering the activity of orthosteric ligands **(**Vavers et al. 2019). The orthosteric binding site of Sig-1R ligands is believed to correspond to the site recognized by (+)-SKF-10,047 and (+)-pentazocine that were the first to be identified as high-affinity selective binders of Sig-1R. Accordingly, allosteric modulators of Sig-1R are defined as compounds that enhance the activity of ligands competing with [^3^H] (+)-pentazocine for receptor binding **(**Cobos et al. 2006). Allosteric modulators of Sig-1R are classified as either positive (PAMs) or negative (NAMs). PAMs enhance the action of orthosteric ligands, whereas NAMs inhibit it. To date, several compounds have been identified as PAMs of Sig-1R, including phenytoin, benzazepine derivatives, stereoisomers of methyphenylpiracetam, and SOMCL-668 **(**Conn et al. 2014). These modulators potentiate the effects of Sig-1R agonists, thereby strengthening their neuroprotective and homeostasis-regulating actions. In contrast, NAMs of Sig-1R have not been reported. Phenytoin, long used as anti-convulsant via sodium channel blockade, was the first compound identified as PAM **(**Merritt and Putnam 1984; Yaari et al. 1986). Radioligand binding studies demonstrated that phenytoin enhanced the binding of [^3^H] dextromethorphan and [^3^H] (+)−3-(3-hydroxyphenyl)-N-propylpiperidine in guinea pig brain, providing the first evidence of Sig-1R allosteric modulation. These findings established phenytoin sensitivity as a hallmark feature of Sig-1R, distinguishing it from Sig-2R. **(**Quirion et al. 1992). SKF83959 (3-methyl-6-chloro-7,8-hydroxy-1-[3-methylphenyl]−2,3,4,5-tetrahydro-1 H-3-benzazepine), an atypical dopamine receptor-1 (D (1)) agonist, was found to act as a PAM by dramatically enhancing the binding of (3)H(+)-pentazocine in brain and liver tissues without affecting Sig-2Rs. This activity may underlie the role of SKF83959 on Sig-1R, which, in turn, may reveal the underlying mechanism for the D (1) receptor-independent effect of the drug **(**Guo et al. 2013; Vavers et al. 2019). Moreover, SKF83959 was also found to enhance the activity of endogenous dehydroepiandrosterone (DHEA) and suppress microglia-mediated inflammation by reducing pro-inflammatory mediators and oxidative stress **(**Wu et al. 2015). SOMCL-668, a selective and potent PAM, ameliorated PCP-induced schizophrenia-like behaviors in mice and reversed PCP-induced reductions in p-AKT/AKT, p-CREB/CREB, and BDNF expression. These effects were blocked by Sig-1R antagonism or gene knockout, confirming receptor involvement and supporting SOMCL-668 potential as a novel therapeutic strategy for psychotic disorders **(**Chen et al. 2022).

## Conclusion

In conclusion, accumulating evidence from clinical and preclinical investigations supports the developing perspective that proteinopathy, marked by disrupted proteostasis, ER stress, and the accumulation of misfolded proteins, significantly contributes to the pathophysiology of schizophrenia. This pathogenic intersection with neurodegenerative processes underscores new treatment options beyond traditional dopaminergic strategies. Sig-1R agonists, due to their distinctive capacity to regulate protein folding, chaperone activity, and cellular stress responses, present significant neuroprotective and homeostatic advantages. Sig-1R agonists offer a promising treatment approach by reestablishing intracellular signaling equilibrium and enhancing cellular resilience to combat the fundamental cellular dysfunctions linked to schizophrenia-related proteinopathy. Additional translational research and clinical trials are necessary to fully exploit their potential in altering disease progression and enhancing cognitive and functional outcomes in schizophrenia.

## Data Availability

No datasets were generated or analysed during the current study.

## Abbreviations

AMPK: AMP-activated protein kinase
ASK1: apoptosis signal-regulating kinase 1
ATF4: activating transcription factor 4
ATF6: activating transcription factor 6
ATGs: autophagy-related genes
Bax: bcl-2- associated X protein
Bcl-2: B-cell lymphoma 2
BDNF: brain-derived neurotrophic factor
bHLH: basic helix-loop-helix
BID: BH3-interacting domain death agonist
BIM: Bcl-2 interacting mediator
BiP: binding immunoglobulin protein
CHOP: C/EBP homologous protein
CNS: central nervous system
CRMP1: collapsin response mediator protein 1
D (1): dopamine-1 receptor
DISC1: disrupted-in-Schizophrenia 1
DTNBP1: dystobrevin-binding protein 1
eIF2α: eukaryotic initiation factor 2α
ER: endoplasmic reticulum
GAD67: glutamate decarboxylase 67
GADD34: DNA damage-inducible protein 34
GRP78: glucose-regulated protein 78
GRP78: 78 kDa glucose-regulated protein
HSPA5: heat shock protein 5
Iba-1: ionized calcium-binding adaptor molecule 1
IP3: inositol 1,4,5-trisphosphate type 3
IRE1: inositol-requiring enzyme type 1
JNK: c-Jun N-terminal protein kinase
MAM: mitochondria-associated membrane
mTOR: mammalian target of rapamycin
NAMs: negative allosteric modulators
NDEL1: nuclear distribution element-like 1
NGF: nerve growth factor
NPAS3: neuronal PAS domain protein 3
NMDAR: N-methyl D-aspartate receptor
nNOS: neuronal nitric oxide synthase
NOS1AP: nNOS adaptor protein
PAMs: positive allosteric modulators
PCP: phencyclidine
PERK: protein kinase R-like ER kinase
PP1: protein phosphatase 1
PSD-95: postsynaptic density protein-95
RIDD: regulated IRE1-dependent decay
ROS: reactive oxygen species
Sig-1R: sigma-1 receptor
SNPs: single nucleotide polymorphism
SSRIs: selective serotonin reuptake inhibitor
TNF-α: tumor necrosis factor-α
TRAF2: tumor necrosis factor (TNF) receptor associated factor-2
TRIOBP: TRIO and F-actin binding protein
ULK1/2: unc-51-like kinases 1 and 2
UPR: unfolded protein response
UPS: ubiquitin-proteasome system
XBP1: X-box-binding protein 1
